# Prediction of death rates for cardiovascular diseases and cancers

**DOI:** 10.1002/cai2.47

**Published:** 2023-02-09

**Authors:** Oleg Gaidai, Yihan Xing, Rajiv Balakrishna, Jiayao Sun, Xiaolong Bai

**Affiliations:** ^1^ Shanghai Engineering Research Center of Marine Renewable Energy, College of Engineering Science and Technology Shanghai Ocean University Shanghai China; ^2^ Department of Mechanical and Structural Engineering and Materials Science University of Stavanger Stavanger Norway; ^3^ School of Naval Architecture & Ocean Engineering Jiangsu University of Science and Technology Zhenjiang China

**Keywords:** cardiovascular disease, cancer, probability forecast, public health, mathematical biology

## Abstract

**Background:**

To estimate cardiovascular and cancer death rates by regions and time periods.

**Design:**

Novel statistical methods were used to analyze clinical surveillance data.

**Methods:**

A multicenter, population‐based medical survey was performed. Annual recorded deaths from cardiovascular diseases were analyzed for all 195 countries of the world. It is challenging to model such data; few mathematical models can be applied because cardiovascular disease and cancer data are generally not normally distributed.

**Results:**

A novel approach to assessing the biosystem reliability is introduced and has been found to be particularly suitable for analyzing multiregion environmental and healthcare systems. While traditional methods for analyzing temporal observations of multiregion processes do not deal with dimensionality efficiently, our methodology has been shown to be able to cope with this challenge.

**Conclusions:**

Our novel methodology can be applied to public health and clinical survey data.

## Graphical abstract

A novel health system reliability method has been developed and applied to cardio and cancer death rate data. Accurate disease multiregional prediction is done.

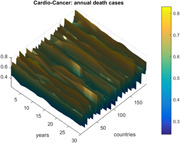

**Abbreviations**
CVD: cardiovascular disease
MDOF: multidegree of freedom

## 1 BACKGROUND

Cardiovascular disease (CVD) refers to a range of diseases affecting the heart and blood vessels including hypertension (high blood pressure), coronary heart disease and heart attacks, cerebrovascular diseases (e.g., stroke and heart failure), and various other heart diseases. Cancers are defined by the National Cancer Institute as diseases in which abnormal cells can divide and infiltrate nearby tissues. Cancers can arise in many parts of the body; thus, there is a wide range of cancer types, as shown below, some of which spread to other parts of the body through the blood and lymph systems. CVD and cancer are the leading causes of death worldwide, therefore analyzing bivariate statistics is important. This study is concerned with public health systems rather than health at the level of the individual. The research is not clinical in nature; the goal is to estimate the burden imposed by CVD and cancer on public health systems in different countries at any given time. We analyze mortality literature data for both CVDs [1, 2, 3, 4, 5, 6, 7, 8] and cancer [9, 10, 11, 12, 13, 14, 15, 16, 17, 18, 19, 20, 21, 22, 23, 24, 25, 26, 27, 28, 29].

Assessing the reliability of healthcare systems and estimating excess mortality from CVDs using conventional statistical methods are challenging [30, 31, 32, 33, 34, 35]. To achieve the latter goal over large areas, degrees of freedom are typically calculated for random variables governing dynamic biological systems. In principle, the reliability of a complex biological system can be accurately estimated if there are sufficient measurements or by using Monte Carlo simulations. For CVDs and cancers, however, data are scarce before 1990 [30]. Against this background, we introduce a novel method for assessing the reliability of biological and healthcare systems, to aid prediction and management of excess mortality from CVD. This study focused on cross‐correlations in CVD and cancer deaths among countries within the same climatic zone. Worldwide health data and related research are readily available online [30].

Lifetime data analysis with the application of extreme value theory is widespread in the fields of medicine and engineering, [30]. A recent paper presented the arguments for and against using the upper distribution of life expectancy data [1]. A bivariate lifetime distribution is often assumed when analyzing statistical data [3]. A new approach that uses Clayton, Gumbel, and inverse Gaussian power variance functions, as well as conditional sampling and numerical approximation, was applied for survival analysis [2]. However, few studies have aimed to predict excess CVD and cancer mortality; this paper aimed to address this deficit.

In this paper, excess mortality from CVD is viewed as an unexpected event that may occur in any country at any time. The nondimensional factor λ is used to predict CVD risk. Biological systems are influenced by environmental parameters that can be modeled as ergodic processes. The CVD and cancer incidence data for 195 countries during the period 1990–2019 were retrieved [30]. The biological system under consideration herein can be regarded as a multidegree of freedom (MDOF) dynamic system with highly interrelated regional components/dimensions. This study focused on predicting excess mortality rather than symptoms.

## 2 METHODS

Consider an MDOF biosystem subjected to random ergodic environmental influences. The other alternative is to view the process as being dependent on specific environmental parameters whose variation in time may be modeled as an ergodic process on its own. The MDOF biomedical response vector process $\vec{R}\equiv R\left(t\right)$ is measured and/or simulated over a sufficiently long time interval (0,T). Unidimensional global maxima over the entire time span (0,T) are denoted as $X_{T}^{\max}=\max_{0\leq t\leq T}X\left(t\right)$, $Y_{T}^{\max}=\max_{0\leq t\leq T}Y\left(t\right)$, $Z_{T}^{\max}=\max_{0\leq t\leq T}Z\left(t\right),\text{…}$. By sufficiently long time T, one primarily means a large value of T with respect to the dynamic system autocorrelation time.

Let $X_{1},\text{…},X_{N_{X}}$ be consequent in the time local maxima of the bioprocess X(t) at monotonously increasing discrete time instants $t_{1}^{X}<\text{…}<t_{N_{X}}^{X}$ in (0,T). The analogous definition follows for other MDOF biological system response components Y(t),Z(t),… with $Y_{1},\text{…},Y_{N_{Y}};$ $Z_{1},\text{…},Z_{N_{Z}}$, and so on. For simplicity, all R(t) components, and therefore, its maxima are assumed to be nonnegative. The aim is to estimate system failure probability

(1) $$\begin{array}{lll} 1-P & = & \text{Prob}(X_{T}^{\max}>\eta_{X}\cup Y_{T}^{\max}\\ & & >\;\eta_{Y}\cup Z_{T}^{\max}>\eta_{Z}\cup \text{…}), \end{array}$$

with

(2) $$\begin{array}{lll} P & = & \overset{\left(\eta_{X},\eta_{Y},\eta_{Z},\text{…}\right)}{\underset{\left(0,0,0,\text{…}\right)}{∭}}p_{X_{T}^{\max},Y_{T}^{\max},Z_{T}^{\max},\text{…}}\\ & & \left(X_{T}^{\max},Y_{T}^{\max},Z_{T}^{\max},\text{…}\right)dX_{T}^{\max}dY_{N_{Y}}^{\max}dZ_{N_{z}}^{\max}\text{…}, \end{array}$$

being the probability of nonexceedance for response components $\eta_{X}$, $\eta_{Y}$, $\eta_{Z}$, … critical values; ∪ denotes logical unity operation «or»; and $p_{X_{T}^{\max},Y_{T}^{\max},Z_{T}^{\max},\text{…}}$ being joint probability density of the global maxima over the entire time span (0,T).

In practice, however, it is not feasible to estimate the latter joint probability distribution directly $p_{X_{T}^{\max},Y_{T}^{\max},Z_{T}^{\max},}\text{…}$ due to its high dimensionality and available data set limitations. In other words, the time instant when either X exceeds, Y exceeds, Z exceeds, and so on, the system is regarded as immediately failed. Fixed failure levels $\eta_{X}$, $\eta_{Y}$, $\eta_{Z}$, … are, of course, individual for each unidimensional response component of R(t). $X_{N_{X}}^{\max}=\max\{X_{j};j=1,\text{…},N_{X}\}=X_{T}^{\max}$, $Y_{N_{Y}}^{\max}=\max\{Y_{j};j=1,\text{…},N_{Y}\}=Y_{T}^{\max}$, $Z_{N_{z}}^{\max}=\max\{Z_{j};j=1,\text{…},N_{Z}\}=Z_{T}^{\max}$, and so on, see Naess and Gaidai [32] and Naess and Moan [49].

Next, the local maxima temporal instants $\left[t_{1}^{X}<\text{…}<t_{N_{X}}^{X};t_{1}^{Y}<\text{…}<t_{N_{Y}}^{Y};t_{1}^{Z}<\text{…}<t_{N_{Z}}^{Z}\right]$ in monotonously nondecreasing order being sorted into one single merged synthetic time vector $t_{1}\leq \text{…}\leq t_{N}$. Note that $t_{N}=\max\{t_{N_{X}}^{X},t_{N_{Y}}^{Y},t_{N_{Z}}^{Z},\text{…}\}$, $N=N_{X}+N_{Y}+N_{Z}+\;\text{…}$. In this case, $t_{j}$ represents the local maxima of one of the MDOF biosystem response components either X(t), Y(t), or Z(t), and so on. That means that having R(t) time record, one just needs to continuously and simultaneously screen for unidimensional response component local maxima and record its exceedance of the MDOF limit vector $\left(\eta_{X},\eta_{Y},\eta_{Z},\ldots \right)$ in any of its components X,Y,Z,…. The local unidimensional response component maxima are merged into one temporal nondecreasing vector $\vec{R}=\left(R_{1},R_{2},\text{…},R_{N}\right)$ in accordance with the merged time vector $t_{1}\leq \text{…}\leq t_{N}$. That is to say, each local maxima $R_{j}$ is the actual encountered local maxima corresponding to either X(t), Y(t), or $Z\left(t\right)$, and so on. Finally, the unified limit vector $\left(\eta_{1},\text{…},\eta_{N}\right)$ is introduced with each component $\eta_{j}$ is either $\eta_{X}$, $\eta_{Y}$, or $\eta_{Z}$ and so on, depending on which of X(t) or Y(t) or $Z\left(t\right)$, and so forth, corresponds to the current local maxima with the running index j.

Next, a scaling parameter 0<λ≤1 is introduced to artificially simultaneously decreases limit values for all biosystem response components, namely, the new MDOF limit vector $\left(\eta_{X}^{\lambda },\eta_{Y}^{\lambda },\eta_{z}^{\lambda },\ldots \right)$ with $\eta_{X}^{\lambda }\equiv \lambda \cdot \eta_{X}$, $\equiv \lambda \cdot \eta_{Y}$, $\eta_{z}^{\lambda }\equiv \lambda \cdot \eta_{Z}$, … is introduced. The unified limit vector $\left(\eta_{1}^{\lambda },\text{…},\eta_{N}^{\lambda }\right)$ introduced with each component $\eta_{j}^{\lambda }$ is either $\eta_{X}^{\lambda }$, $\eta_{Y}^{\lambda }$, or $\eta_{z}^{\lambda }$ and so on. The latter automatically defines probability P(λ) as a function of λ; note that P≡P(1) from Equation (1). Nonexceedance probability P(λ) can be now estimated as follows:

(3) $$\begin{array}{lll} P\left(\lambda \right) & = & \text{Prob}\left\{R_{N}\leq \eta_{N}^{\lambda },\text{…},R_{1}\leq \eta_{1}^{\lambda }\right\}\\ & = & \text{Prob}\left\{R_{N}\leq \eta_{N}^{\lambda }|R_{N-1}\leq \eta_{N-1}^{\lambda },\text{…},R_{1}\leq \eta_{1}^{\lambda }\right\}\\ & & \cdot \text{Prob}\left\{R_{N-1}\leq \eta_{N-1}^{\lambda },\text{…},R_{1}\leq \eta_{1}^{\lambda }\right\}\\ & = & \prod_{j=2}^{N}P\text{rob}\left\{R_{j}\leq \eta_{j}^{\lambda }|R_{j-1}\leq \eta_{1j-}^{\lambda },\text{…},R_{1}\leq \eta_{1}^{\lambda }\right\}\\ & & \cdot \text{Prob}\left(R_{1}\leq \eta_{1}^{\lambda }\right). \end{array}$$

In practice, the dependency between neighboring $R_{j}$ values is not always negligible; thus, the following one‐step (i.e., “conditioning level”; k=1) memory approximation is introduced

(4) $$\begin{array}{l} \text{Prob}\left\{R_{j}\leq \eta_{j}^{\lambda }|R_{j-1}\leq \eta_{j-1}^{\lambda },\text{…},R_{1}\leq \eta_{1}^{\lambda }\right\}\\ \;\approx \text{Prob}\left\{R_{j}\leq \eta_{j}^{\lambda }|R_{j-1}\leq \eta_{j-1}^{\lambda }\right\}, \end{array}$$

for 2≤j≤N (called here conditioning level k=2). Approximation being introduced by Equation (4) may be further expressed as

(5) $$\begin{array}{l} \text{Prob}\left\{R_{j}\leq \eta_{j}^{\lambda }|R_{j-1}\leq \eta_{j-1}^{\lambda },\text{…},R_{1}\leq \eta_{1}^{\lambda }\right\}\\ \;\approx \text{Prob}\left\{R_{j}\leq \eta_{j}^{\lambda }|R_{j-1}\leq \eta_{j-1}^{\lambda },R_{j-2}\leq \eta_{j-2}^{\lambda }\right\}, \end{array}$$

where 3≤j≤N (will be called conditioning level k=3) and so on. The motivation is to monitor each independent failure that happened locally first in time, thus avoiding cascading local intercorrelated exceedances [36, 37, 38, 39, 40, 41, 42, 43, 44, 45, 46, 47, 48].

Equation (5) presents subsequent refinements of the statistical independence assumption. The latter type of approximations enables capturing the statistical dependence effect between neighboring maxima with increased accuracy. Since the original MDOF bioprocess R(t) was assumed ergodic and therefore stationary, probability $p_{k}\left(\lambda \right)≔\text{Prob}\{R_{j}>\eta_{j}^{\lambda }|R_{j-1}\leq \eta_{j-1}^{\lambda },R_{j-k+1}\leq \eta_{j-k+1}^{\lambda }\}$ for j≥k will be independent of j but only dependent on conditioning level k. Thus, the nonexceedance probability can be approximated as in the Naess–Gaidai method, see [32, 49], where:

(6) $$P_{k}(\lambda )\approx \text{exp}\left(-{N\cdot p}_{k}\left(\lambda \right)\right),k\geq 1.$$

Note that Equation (6) follows from Equation (1) by neglecting $\text{Prob}\left(R_{1}\leq \eta_{1}^{\lambda }\right)\approx 1$, as the design failure probability is usually very small. Further, it is assumed that N≫k. Note that Equation (5) is similar to the well‐known mean up‐crossing rate equation for the probability of exceedance [32, 49]. There is observed convergence with respect to conditioning parameter k

(7) $$P=\lim_{k\to \infty }P_{k}(1);p\left(\lambda \right)=\lim_{k\to \infty }p_{k}\left(\lambda \right).$$

Note that Equation (6) for k=1 turns into the quite well‐known nonexceedance probability relationship with the mean up‐crossing rate function

(8) $$P\left(\lambda \right)\approx \text{exp}\left(-\nu^{+}(\lambda )T\right);\left(\lambda \right)=\int_{0}^{\infty }\zeta p_{R\dot{R}}\left(\lambda ,\zeta \right)d\zeta ,$$

where $\nu^{+}(\lambda )$ is the mean up‐crossing rate of the response level λ for the above assembled nondimensional vector R(t) assembled from scaled MDOF biosystem response $\left(\frac{X}{\eta_{X}},\frac{Y}{\eta_{Y}},\frac{Z}{\eta_{Z}},\text{…}\right)$. The proposed methodology can also treat nonstationary cases. An illustration of how the methodology can be used to treat nonstationary cases is provided as follows. Consider a scattered diagram of m=1,…,M bioenvironmental states, with each short‐term bioenvironmental state having probability $q_{m}$ so that $\sum_{m=1}^{M}q_{m}=1$. The corresponding long‐term equation is then

(9) $$p_{k}(\lambda )\equiv \sum_{m=1}^{M}p_{k}\left(\lambda ,m\right)q_{m},$$

with $p_{k}(\lambda ,m)$ being the same function as in Equation (7) but corresponding to a specific short‐term environmental state with the number m. Note that this statistical model has already been validated [47, 50, 51, 52].

## 3 RESULTS

Prediction of CVD and cancer has long been a target in the fields of epidemiology and mathematical biology. Public health systems are dynamic, highly nonlinear, multidimensional, and spatially diverse systems that are challenging to analyze. Previous studies have used a variety of approaches to predict CVD and cancer cases. In this section, the above‐described methodology is applied to real‐world CVD data sets for all countries of the world.

The statistical data in the present section are from the “Our World in Data” website [30], which provides annual CVD death rates for all countries for the period 1990–2019. The death rates for the 195 countries (components X,Y,Z…) constitute 195 dimensional (195D) data for a dynamic biological system.

General failure limits ($\eta_{X},\eta_{Y},\eta_{Z},\text{…}$), that is, CVD thresholds, are less intuitive than setting failure limits for each individual country according to its population, such that X,Y,Z,… are equal to the annual death rate of a given country. The death rate for cancer is lower than that for CVD, but it is typically more painful to die from cancer. In this paper, the “failure limit” for cancer is lowered fourfold to match that for CVD.

Next, the local maxima from all nondimensionalized time series data are merged into a single time series using Equation (5):

(10) $$\begin{array}{lll} \vec{R} & = & (\{\max\left\{X_{1}^{\text{cardio}},X_{1}^{\text{cancer}}\right\},\max\left\{Y_{1}^{\text{cardio}},Y_{1}^{\text{cancer}}\right\},\\ & & \max\left\{Z_{1}^{\text{cardio}},Z_{1}^{\text{cancer}}\right\},\text{…}\},\text{…},\{\max\left\{X_{N}^{\text{cardio}},X_{N}^{\text{cancer}}\right\},\\ & & \max\left\{Y_{N}^{\text{cardio}},Y_{N}^{\text{cancer}}\right\},\max\left\{Z_{N}^{\text{cardio}},Z_{N}^{\text{cancer}}\right\},\text{…}\}). \end{array}$$

Each maximum, such as $\max\{X_{j}^{\text{cardio}},X_{j}^{\text{cancer}}\}$, is inserted into single time series according to its temporal occurrence (denoted by subscript j).

**Figure 1:**
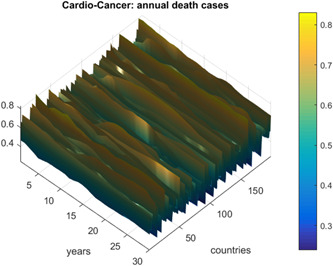
Annual deaths from cardiovascular disease and cancer as a percentage of the population for 195 countries.

**Figure 2:**
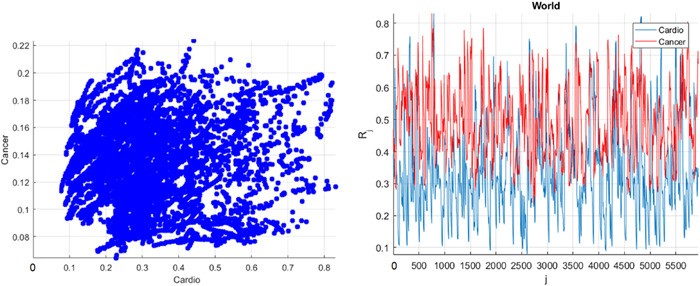
Left: Cross‐correlations between cardiovascular disease (CVD) and cancer cases as a percentage of the population. Right: Annual death rates as a 195‐dimensional vector $\vec{R}$, as a percentage of the population of the corresponding country. The cancer rate was increased fourfold to match that of CVD.

Figure 1 presents the annual deaths from CVD and cancer by country and year. Figure 2 presents the number of new deaths as a 195D vector $\vec{R}$. Data for Uzbekistan were excluded from the analysis because they were regarded as outliers. $\vec{R}$ was assembled from different regional components, that is, CVD data sets. Index j is a running index of local maxima encountered in the “non‐decreasing” time series.

Overall, there is a clear East–West divide in the CVD death rates. Rates across North America and Western/Northern Europe tended to be lower than those across Eastern Europe, Asia, and Africa. For most of Latin America, the rates were moderate. As an example, in France, the age‐standardized CVD death rate was around 86 per 100,000 in 2017, while across Eastern Europe, it was around five times higher (400–500 per 100,000). Uzbekistan had the highest rate of 724 per 100,000.

**Figure 3:**
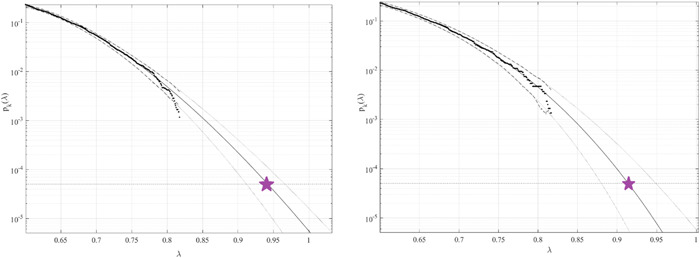
Death rate predictions over 100 years extrapolated from $p_{k}\left(\lambda \right)$. The critical level is indicated by a star. The 95% confidence intervals are indicated by dotted lines. The percentage of the population is represented by the horizontal axis. Left: Predictions based on 30 years of data; Right: predictions based on 15 years of data.

Figure 3 presents the predicted annual CVD death rates (percentage relative to the entire population of a given country) over 100 years, extrapolated from Equation (10). λ=0.6% was used as a cut‐off value. The 95% confidence intervals (CIs) were calculated. According to Equation (5), p(λ) is directly related to the target failure probability (1−P) derived from Equation (1). Therefore, system failure probability can be estimated as $1-P\approx {1-P}_{k}\left(1\right)$. Note that, in Equation (6), N corresponds to the total number of local maxima in response vector $\vec{R}$. Conditioning parameter k=3 was found to be sufficient because of the convergence of k (see Equation 6). In Figure 3, the 95% CIs are relatively narrow, which represents an advantage of the proposed method. Table 1 compares 100‐year predictions based on data for 15‐ and 30‐year periods. The 15‐year data set was derived from the full 30‐year data set by omitting odd years. The 95% CIs were wider for the truncated data set, as expected.

**Table 1:** Predicted cardiovascular disease death rates over 100 years based on 30‐ and 15‐year data sets.

	Predicted death rate (%)	95% CI, lower bound	95% CI, upper bound
30‐year data set	0.942	0.909	0.966
15‐year data set	0.914	0.879	0.949

Abbreviation: CI, confidence interval.

The predicted average annual CVDs over the next 100 years, among all years and countries, were found below 1%. Our methodology uses available data efficiently by assuming that healthcare system data sets are multidimensional and extrapolates death rates even when the data set is relatively limited. The predicted nondimensional factor λ, indicated by the star in Figure 3, represents the probability of excess CVD mortality for any given country. Our method could be applied to predict cancer clusters, rather than merely death rates over time, which would be of high practical importance.

## 4 CONCLUSIONS

Traditional methods for assessing the reliability of healthcare systems on the basis of time series data do not efficiently deal with systems characterized by high dimensionality and cross‐correlations. The main advantage of our methodology is its ability to assess the reliability of high‐dimensional nonlinear dynamic systems. Despite its simplicity, the novel multidimensional modeling strategy introduced herein can be used for accurate forecasting of CVD death rates in individual countries.

We analyzed 195D data, that is, CVD and cancer death rates for 195 countries worldwide, for the period 1990–2019. A novel method for analyzing the reliability of a multidimensional biosystem was applied and the mechanisms of the proposed method were described in detail. Direct measurements and Monte Carlo simulations are both suitable for assessing the reliability of dynamic biological systems; however, the complexity and high dimensionality of such systems necessitate the further development of robust and accurate techniques that can use limited data sets in an efficient manner.

This study predicted an average annual death rate for CVD over a 100‐year period of about 1% across countries and years. Under current national health management approaches, CVDs will continue to represent a threat to the health of the world population.

This study introduced a general‐purpose, robust, and easy‐to‐apply method for analyzing the reliability of multidimensional systems. The method has previously been validated by application to a wide range of simulation models but only in the context of one‐dimensional systems; in general, highly accurate predictions were obtained. Both measurement and numerically simulated time series data can be analyzed. Applying the method to the data set used in this study yielded reasonable confidence intervals, indicating that it could serve as a useful tool for reliability studies of various nonlinear dynamic biological systems. Finally, the suggested methodology has many potential public health applications beyond the prediction of CVD death rates.

## AUTHOR CONTRIBUTIONS

**Oleg Gaidai**: Conceptualization (equal). **Yihan Xing**: Validation (equal). **Rajiv Balakrishna**: Investigation (equal). **Jiayao Sun**: Methodology (equal). **Xiaolong Bai**: Validation (equal).

## CONFLICT OF INTEREST STATEMENT

The authors declare no conflict of interest.

## ETHICS STATEMENT

Not applicable.

## INFORMED CONSENT

Not applicable.

## Data Availability

Data sets analyzed during the current study are available online at https://ourworldindata.org/causes-of-death (“Our World in Data” [30]).
